# Testing of Different Digestion Solutions on Tissue Samples and the Effects of Used Potassium Hydroxide Solution on Polystyrene Microspheres

**DOI:** 10.3390/toxics11090790

**Published:** 2023-09-19

**Authors:** Liesa Geppner, Jakob Karaca, Wencke Wegner, Moritz Rados, Tobias Gutwald, Philemon Werth, Maja Henjakovic

**Affiliations:** 1 Department of Medicine, Faculty of Medicine and Dentistry, Danube Private University, Steiner Landstraße 124, 3500 Krems, Austria; 2 Natural History Museum Vienna, Burgring 7, 1010 Vienna, Austria

**Keywords:** microplastics, 1 µm polystyrene particles, organ samples, enzymatic and alkaline digestion, method development

## Abstract

Microplastic particles are ubiquitous in our environment, having entered the air, the water, the soil, and ultimately our food chain. Owing to their small size, these particles can potentially enter the bloodstream and accumulate in the organs. To detect microplastics using existing methods, they must first be isolated. The aim of this study was to develop a non-destructive method for efficiently and affordably isolating plastic particles. We investigated the digestion of kidney, lung, liver, and brain samples from pigs. Kidney samples were analyzed using light microscopy after incubation with proteinase K, pepsin/pancreatin, and 10% potassium hydroxide (KOH) solution. Various KOH:tissue ratios were employed for the digestion of lung, liver, and brain samples. Additionally, we examined the effect of 10% KOH solution on added polystyrene microplastics using scanning electron microscopy. Our findings revealed that a 10% KOH solution is the most suitable for dissolving diverse organ samples, while enzymatic methods require further refinement. Moreover, we demonstrated that commonly used 1 µm polystyrene particles remain unaffected by 10% KOH solution even after 76 h of incubation. Digestion by KOH offers a simple and cost-effective approach for processing organ samples and holds potential for isolating plastic particles from meat products.

## 1. Introduction

Microplastic pollution is increasingly on the rise and an expanding shift from macro- to microparticles can be expected [1]. Two different sources for the formation of microplastics have been described. Primary microplastics are small plastic particles that are intentionally manufactured to be used in various products such as cosmetics (e.g., microbeads), cleaning products, and industrial abrasives (e.g., pellets and flakes) [2]. Secondary sources include the breakdown and fragmentation of larger plastic debris and the release of microplastics as a result of chemical, physical, and biological degradation processes [3]. Microplastics have been detected in various settings, including the air, oceans, soils, and, accordingly, food items [4,5,6]. Regardless of the source, the ingestion of microplastics can result in adverse effects on human health, triggering a series of biological responses [7].

Regarding the size definition for microplastics, an upper size limit of microplastic particles has been set at 5 mm [8], with 1 µm representing the lower limit [9], and particles of up to 0.1 µm are referred to as submicroplastics [10].

A recent review has emphasized that the prevalence of microplastics in the environment, in drinking water, and especially in human food presents a growing concern [11]. Therefore, developing a simple and efficient method for detecting microplastics in human fluids, human tissue, and various food items, such as meat products, is of the utmost importance. There are already numerous methods for analyzing larger microplastic particles in environmental samples. Microscopy is a commonly used method that allows the visual identification and quantification of microplastics [12].

In order to be able to identify plastic particles using existing spectrometric techniques such as infrared and micro-Raman spectroscopy, as well as mass-based techniques like Pyrolysis-GC-MS, it is necessary to isolate the particles from the biological matrix [10]. Environmental scientists have developed a variety of techniques for separating plastic from organic material, such as sewage sludge, sediment, and water. The most commonly used methods involve acidic and alkaline treatments, although some researchers have also explored enzymatic approaches [13,14,15]. Different experimental setups with similar chemical or enzymatic digestion mixtures have been used for human or animal blood or tissue. Leslie et al. used enzymatic digestion with proteinase K to detect microplastics in the blood for the first time [16].

Nano- and microplastic particles can accumulate in the organs or be cleared into interstitial space due to the cross-linking of vessels, resulting in a reduction in vessel lumina. This has been confirmed with the detection of plastic particles in various human organs. Recent studies have demonstrated the presence of microplastics in the lungs [17] as well as in the placenta [18]. Similarly to what has already been observed in humans, the accumulation of microplastics in the organs of farm animals, such as pigs, cattle, and poultry, is also possible [19]. In experimental settings, the accumulation of plastic has also been detected in various tissues of mice, such as the kidney, lung, liver, and brain. Plastic accumulation and toxicological effects were detected in these animal experiments after application to tissues [20,21]. However, the application amount was partially higher than the physiological concentration that has already been detected in human tissues.

The objective of this study was to adapt and optimize a rapid and efficient protocol for digesting various organ samples without destroying microplastic particles. For this purpose, enzymatic (proteinase K, pepsin, and pancreatin) protocols were compared to an alkaline (10% potassium hydroxide, KOH) digestion treatment on different porcine tissues. These tissues were chosen because they are highly similar to human tissues, and the internal organs of the pig, like the liver, are frequently consumed or used as ingredients in meat specialties.

The uniqueness of proteinase K lies in its ability to degrade a wide range of proteins, including those with stable or hard-to-reach structures. For this reason, proteinase K is employed in numerous molecular biological and histological procedures and has also been used for isolating microplastics from human blood [16]. The objective here was to investigate whether the proteinase K protocol is suitable for the complete digestion of various porcine organs.

In biomedical research, various protocols have been developed for in vitro digestion utilizing the digestive enzymes pepsin and pancreatin. Examples include the digestion of microalgae, bovine, and porcine muscle tissue [22,23,24]. The aim of this study was to employ a straightforward in vitro digestion protocol with pepsin and pancreatin to achieve the complete digestion of porcine organs.

For the isolation of plastic particles from environmental, animal, and human samples, acids and alkalis have frequently been employed, providing a simple and cost-effective method. A 10% KOH (1.8 M) solution, for instance, has been utilized for the dissolution of human placentas [18]. Some studies have indicated that a 10% KOH solution will reduce the recovery of certain plastics, such as polycarbonate, polyvinyl chloride, and polyethylene terephthalate, while others have ruled out degrading effects at milder temperatures [25,26,27,28]. Following extensive literature research that took into account observed effects on various types of plastics, as well as the conduction of numerous preliminary experiments, the alkaline dissolution of kidney, liver, lung, and brain samples in a 10% KOH solution at 37 °C was planned for investigation.

Additionally, the effects of KOH on 5 µm and especially 1 µm polystyrene (PS) microspheres were examined under specific experimental conditions. These model particles were roughly the size of microplastic particles that can circulate in the bloodstream. Polystyrene particles have been recognized as one of the most encountered plastics in human blood [16], are commonly utilized in research studies [20,21], and, due to their extensive surface area, offer a substantial site of interaction for reagents, including the KOH solution in this case. Viewed critically, this does not permit the exclusion of the effects of KOH on various types of plastic particles; it exclusively addresses alterations in PS microspheres. Moreover, the impact of KOH on PS particles actually present in organs could yield different outcomes depending on their properties.

## 2. Materials and Methods

### 2.1. Sample Collection

Human blood samples were acquired from four anonymized healthy donors, comprising one female and three males aged between 22 and 26 years. The samples were collected using 10 mL EDTA Vacutainers (367525, BD Biosciences, Vienna, Austria). Venipuncture was performed with a sterile Vacutainer-Safety-Lok 21G (367282, BD Biosiences). Immediately after sampling, the blood samples were stored in a −20 °C freezer until analysis.

Porcine tissue, including kidney, liver, lung, and brain tissue, was obtained from a local butcher. Upon arrival, the porcine tissue was carefully washed and then cut into smaller portions under sterile conditions to prevent contamination. Each tissue portion was then stored at −20 °C until further use.

### 2.2. Polystyrene (PS) Polymer Microspheres

The used polystyrene (PS) polymer microspheres with sizes of 1 µm and 5 µm had no surface modifications (BS-Partikel GmbH, Mainz, Germany; HS0100-20 and HS0500-20, respectively). These microspheres were produced by the manufacturer using standardized methods and underwent characterization. For the 1 µm microspheres, the manufacturer specified a mean diameter of 0.988 µm with a relative standard deviation (CV) of 2.2%, while the 5 µm microspheres had a mean diameter of 4.96 µm with a CV of 1.6%. The microspheres were composed of poly(styrene-co-divinylbenzene), possessed a density of 1.05 g/cm^3^, and exhibited a refractive index of 1.59 at 25 °C.

### 2.3. Microscopy

A light microscope (Leica DM2500 LED, Leica Microsystems AG, Balgach, Switzerland) was used for the visualization of alkaline and enzyme-dependent digestion and the counting of polystyrene (PS) plastic particles. Unless otherwise stated, pictures were taken with fortyfold magnification.

Scanning electron microscopy (SEM) was performed at the Central Research Laboratories of the Natural History Museum of Vienna (NHMW). The samples were first coated with platinum (Leica EM CSD 500, Leica Microsystems AG, Balgach, Switzerland) and subsequently visualized with a JEOL JSM-6610 (JEOL AG, Freising, Germany) at an accelerating voltage of 20 kV and a spot size of 40.

### 2.4. Digestion

#### 2.4.1. Enzymatic Digestion Method Using Proteinase K

For enzymatic digestion using proteinase K, the digestion protocol for blood described by Leslie et al. was adopted [16]. The frozen tissue (kidney, lung, liver, brain) was mechanically processed using a mortar until a homogeneous pulpy consistency was achieved. The amount of tissue was adjusted to the hematocrit value. Accordingly, 0.45 g of homogenized tissue, which approximately corresponded to the cellular component of 1 g of blood, was used. Additionally, a control setup was performed with 1 g of blood.

Then, 15 mL of Tris-HCl buffer (400 mM Tris-HCL; J22638.K2; Thermo Fisher Scientific, Vienna, Austria; pH 8; 0.5% SDS ultrapure; 2326.1; Carl Roth GmbH + Co. KG, Karlsruhe, Germany) was added per 1 g of blood or 0.45 g of homogenized tissue and incubated in a water bath at 60 °C for 1 h to denature the proteins. For further digestion, 100 µL of proteinase K (1 mg/mL; ≥3.0 unit/mg solid; P8044; Sigma-Aldrich Handels Gmbh, Vienna, Austria) was inserted together with 1 mL of 5 mM calcium chlorite (CaCl_2_; HN04.2; Carl Roth) and incubated for another 2 h at 50 °C. The flask containing these elements was then shaken for 20 min at room temperature and finally heated in a water bath to 60 °C for another 20 min.

The effects of proteinase K on blood and tissue samples were investigated using light microscopy (Leica DM2500 LED, Leica Microsystems AG, Balgach, Switzerland) in a minimum of ten pictures, and the amount of cell debris was determined using Fiji (ImageJ, version 2.1.0/1.53c; National Institutes of Health). In order to compare the results of the blood digestion with proteinase K with the conditions of an initial time point, the blood was diluted with PBS according to the work volume in the proteinase K digestion protocol.

#### 2.4.2. Enzymatic Digestion Method Using Pepsin and Pancreatin

The porcine pepsin (KK38.1; Carl Roth GmbH + Co. KG, Karlsruhe, Germany) had a specific enzyme activity of ≥2000 FIP-U/g as provided by the manufacturer. For the stock solution, the pepsin was dissolved in 10 mM HCl to obtain a concentration of 2 mg/mL.

The porcine pancreatin (A0585; AppliChem GmbH, Darmstadt, Germany) had a specific enzyme activity of 36,000 FIP-U/g as provided by the manufacturer. For the stock solution, the pancreatin was dissolved in Dulbecco’s phosphate-buffered saline (DPBS; 14190-144; Thermo Fisher Scientific) to obtain a concentration of 2 mg/mL.

The porcine kidney, lung, liver, and brain tissue was mechanically processed using a mortar until a homogeneous pulpy consistency was achieved. An amount of 0.45 g of homogenized tissue was used for each experiment.

The first step of the enzymatic digestion protocol was the denaturation and pre-digestion of the homogenized tissue by hydrochloric acid (HCl) and pepsin. For the optimal activity of the pepsin, homogenized tissue with an added HCl solution (160 mM in end volume) was mixed in 100 mL Erlenmayer flasks and pre-incubated in a water bath for 5 min at 37 °C before 2 mg of pepsin was added. The mixture was incubated for 4 h at 37 °C in a water bath with shaking.

Subsequently, the mixture was buffered to a pH between 7 and 7.4 by adding 1 M sodium hydrogen carbonate (NaHCO_3_; 8551.1; Carl Roth GmbH). After neutralization, 20 mg of pancreatin was added as a single dose and incubated for 24.5 h.

Enzymatic digestion was investigated using light microscopy (Leica DM2500 LED) after a 4 h incubation time for pepsin and a 24.5 h incubation time for pancreatin, with a minimum of twenty pictures across two cover slips per sample. The amount of cell debris was determined using Fiji (ImageJ, version 2.1.0/1.53c; National Institutes of Health).

#### 2.4.3. Alkaline Digestion Method

For alkaline digestion, porcine organs (kidney, lung, liver, brain) were treated with 10% (1.8 M) potassium hydroxide solution (KOH; P747.1; Carl Roth). The ratios of sample to alkaline reagent used were 1:4 (*w*/*v*) for the first experimental approach and 1:8 (*w*/*v*) for a further tissue digestion trial. First, KOH solutions were transferred to autoclaved 100 mL Erlenmeyer flasks; then, small-cut pieces of different tissues were added. The flasks were incubated in a water bath at 37 °C for 76 h. The digestion progress was checked using light microscopy at the time points of 0 h (immediately after preparing the mixture), 6 h, 24 h, and 76 h for the 1:4 (*w*/*v*) ratio. An evaluation of the digestive process was conducted solely after durations of 6 h and 76 h for the 1:8 (*w*/*v*) ratio.

The effects of alkaline reagents on various porcine tissues were investigated using light microscopy (Leica DM2500 LED) in a minimum of twenty pictures across two cover slips per sample, and the amount of cell debris was determined using Fiji (ImageJ, version 2.1.0/1.53c; National Institutes of Health).

### 2.5. Effects of Digestion Protocols on Plastic Particles

In addition, the effect of 10% KOH digestion solution on 1 µm and 5 µm polystyrene (PS) particles was examined.

For this purpose, 1 µm and 5 µm PS particles were added to alkaline digestion solution. To prevent the particles from being obscured by digestive residues or aggregating to them, the tissue samples were replaced with H_2_O and 1 µm particles were investigated at a 1:4 ratio, while 5 µm particles were investigated at a 1:8 ratio with KOH. Only particle-free water was used for the alkaline control. The solutions were then incubated for 76 h at 37 °C. Light microscopy was used to directly examine the particles in the incubation solution at different time points between 0 h (immediately after preparing the mixture) and 76 h.

In addition, effects on the size as well as changes in the surface structure of the 1 µm isolated particles from the alkaline digestion solution and the alkaline control group were investigated with SEM (JEOL JSM-6610). For this purpose, the samples were subsequently filtered using 0.45 µm track-etched polycarbonate membrane filters (A046.1; Carl Roth). The filters were washed three times with 15 mL of particle-free H_2_O, dried, and then affixed to the corresponding SEM carbon tape.

### 2.6. Statistical Analysis

All data are represented as means ± SD. Statistical analyses were performed using a two-tailed unpaired *t*-test when testing between two time points, and one-way analysis of variance with Dunnett’s multiple comparison test was performed for within-group comparisons (GraphPad Prism9, version 9.3.1). Statistical significance was set at *p* < 0.05.

## 3. Results

### 3.1. Kidney, Lung, Liver, and Brain Tissue Digestion Using Proteinase K

**Figure 1:**
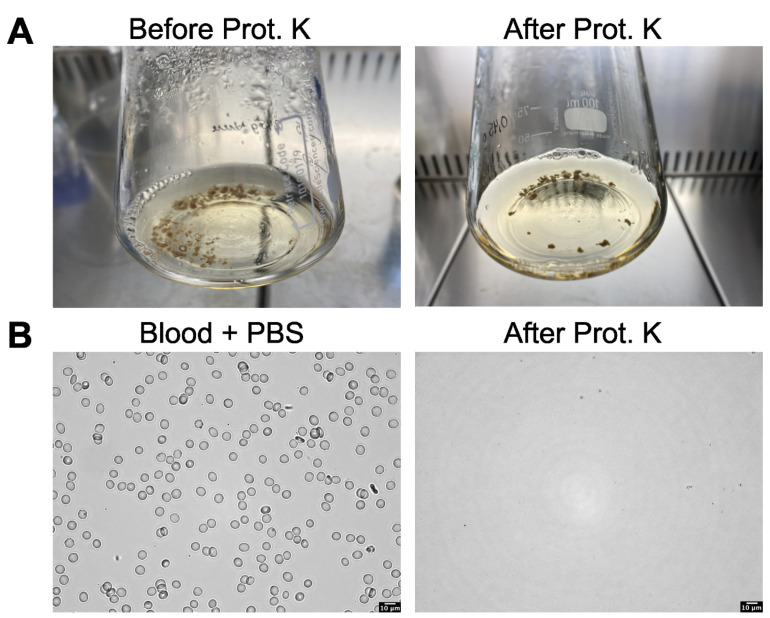
Enzymatic digestion of porcine kidney samples (**A**) and blood (**B**) using proteinase K (Prot. K). (**A**) These images depict homogenized kidney tissue in the digestion solution before and after incubation in proteinase K, as microscopic examination was not feasible. (**B**) These representative microscopic images show a blood sample diluted in PBS and after digestion with Proteinase K.

The enzymatic digestion method with proteinase K was applied to homogenized kidney, lung, liver, and brain tissue according to the protocol of Leslie et al. [16]. Almost no digestive progress could be observed when 1 g of kidney tissue was used. In reducing the amount of tissue to approximately the hematocrit value of the blood, slight digestive progress could be seen. Figure 1A shows that fewer kidney tissue residues were visible after complete proteinase K digestion compared to protein denaturation before proteinase K. However, even with these adjustments, sufficient digestion was not yet to be observed. Similarly, the lung, liver, and brain samples exhibited only minimal digestion upon treatment with proteinase K (Figure S1). Blood digestion with proteinase K gives very good and highly significant results, as can be seen in the experiment of Leslie et al. [16] and in our experiment (Figure 1B). Compared with an adequately diluted blood sample in PBS, only some, almost invisible, cellular debris remained after the digestion with proteinase K (Figure 1B).

### 3.2. Kidney, Lung, Liver, and Brain Tissue Digestion Using Pepsin and Pancreatin

As a further manner of tissue digestion, an enzymatic method utilizing pepsin and pancreatin was employed. After incubation with pepsin, the full amount of pancreatin was added, and microscopic images were taken at specific time intervals. There was one microscopic time point 4 h after the addition of the pepsin, and a second microscopic time point was performed 24.5 h after the complete addition of the pancreatin.

**Figure 2:**
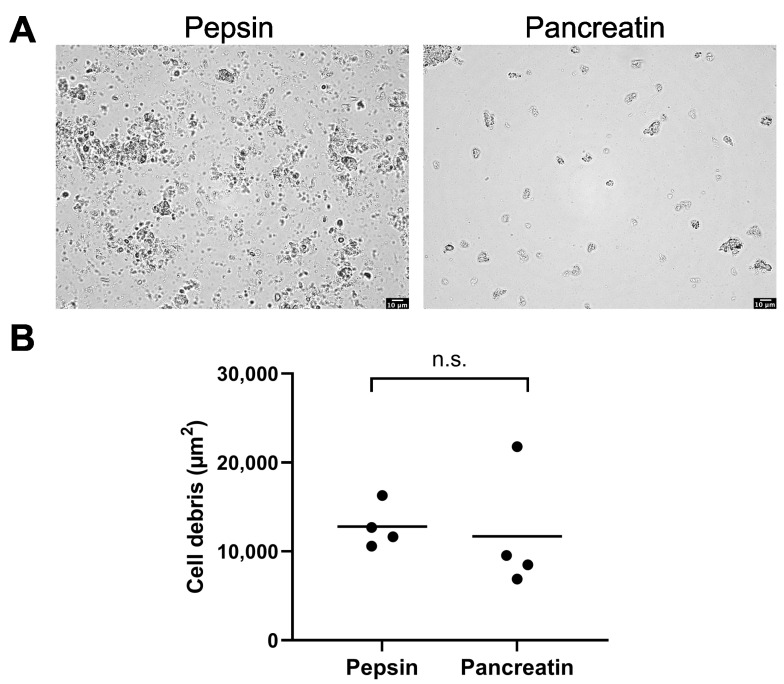
Enzymatic digestion of kidney tissue using pepsin and pancreatin. The kidney homogenates were incubated for 4 h in a 2 mg/mL pepsin solution, followed by an additional 24.5 h in a 2 mg/mL pancreatin solution at 37 °C. (**A**) These representative microscopic images show the progress of digestion after incubation in pepsin and after subsequent incubation in pancreatin. (**B**) Cell debris was detected with light microscopy, and subsequent image analysis used a Fiji macro. Data points represent experimental repetition, and the crossbar represents the mean. *n* = 4. Statistical significance was determined using a two-tailed unpaired *t*-test. n.s.: not significant.

After 4 h of incubation of homogenized kidney tissue with pepsin, large areas of cell debris remained visible (Figure 2A). However, after pancreatin incubation, the kidney tissue exhibited the presence of conspicuous large cell debris, which remained observable under microscopic examination (Figure 2A).

Only minimal differences were observed between the effects of pepsin and pancreatin on kidney digestion (Figure 2B). This observation contrasts with Figure 2A, in which the undigested area appears to have decreased markedly after 24.5 h of incubation with pancreatin. The reason for this apparent inconsistency is that in Figure 2B, the value of cell debris for incubation in pancreatin has been corrected by a factor of 5.7 to account for the greater volume during pancreatin digestion compared with that after 4 h of pepsin incubation. Similarly, it was not possible to digest lung, liver, or brain tissues using pepsin or pancreatin (Figure S2).

### 3.3. Kidney, Lung, Liver, and Brain Tissue Digestion Using KOH

**Figure 3:**
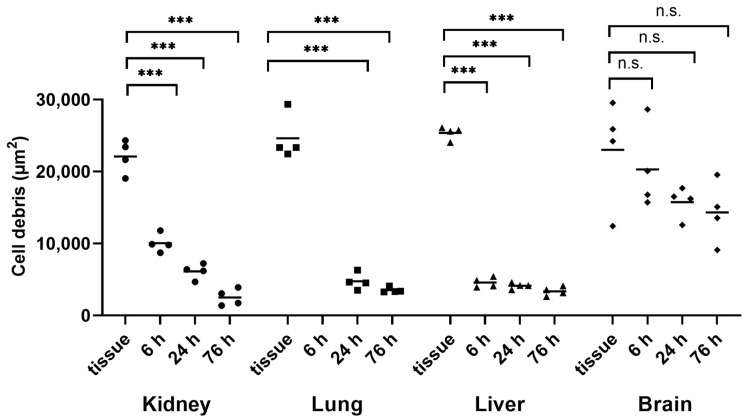
Time-dependent alkaline digestion of tissue samples using 10% KOH at a ratio of 1:4 (*w*/*v*) and an incubation temperature of 37 °C. This graphic illustration shows kidney, lung, liver, and brain tissue digestion. Cell debris was detected with light microscopy, and subsequent image analysis was performed with a Fiji macro. Data points represent experimental repetition, and the crossbar represents the mean. *n* = 4. Statistical significance was determined using a one-way analysis of variance with Dunnett’s multiple comparison test for within-group comparisons (20 µm thickened cryostat versus 6 h, 24 h and 76 h effect of 10% KOH solution on tissue samples). n.s.: not significant; ***: *p* < 0.001.

In addition, an alkaline method using potassium hydroxide (KOH) was tested to verify the reliable and sufficient digestion of kidney tissue. Therefore, small kidney tissue pieces were first treated with a 10% KOH solution in a 1:4 (*w*/*v*) ratio, and the amount of cell debris was analyzed using light microscopy. Figure 3 shows that a highly significant progress in digestion could already be seen after 6 h. After 76 h, the area of undigested cell debris had already decreased to 11.3 ± 4.6% compared to the kidney tissue section, which can be assumed to have been at 100 ± 9.1%.

Due to the fact that the digestion provided good results for the kidney tissue, further tissues, such as the lung, liver, and brain, were also tested at a 1:4 (*w*/*v*) tissue-to-KOH ratio.

Both the lung tissue and the liver tissue showed similarly good results compared to the kidney tissue, with undigested cell remains areas of 14.2 ± 1.4% and 13.2 ± 2.1%, respectively (Figure 3). However, in the case of the lung tissue, after 6 h, there were still small tissue fragments visible in the solution, which is why the microscopic analysis was not considered meaningful at that point. The brain tissue provided the worst digestion results. Even after 76 h of incubation in 10% KOH solution, the sample showed 62.2 ± 16.2% of undigested residues, which indicated no significant differences compared to the brain tissue section (100 ± 27.9%).

### 3.4. Effect of 10% KOH Solution on Polystyrene (PS) Microspheres

**Figure 4:**
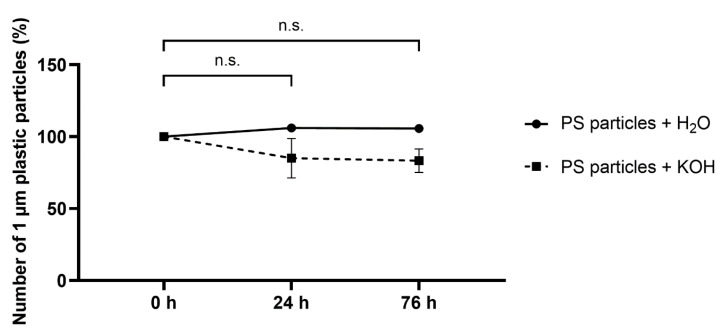
Effect of KOH solution on 1 µm polystyrene (PS) plastic particles. PS particles were incubated with 10% KOH solution in the absence of human blood and in an H_2_O control at 37 °C for up to 76 h. They were counted using the Fiji cell counter tool. Data represent means ± SD. *n* = 3 per treatment. n.s.: not significant; within the KOH group in comparison with 0 h.

The effect of the KOH solution on 1 µm polystyrene particles (PS) was tested. For this experiment, the 1 µm PS particles were incubated in 10% KOH solution and in H_2_O as control attempt for 0 h, 24 h, and 76 h at 37 °C. However, at 37 °C, the used 1 µm particles in the KOH did not show any significant differences (Figure 4).

**Figure 5:**
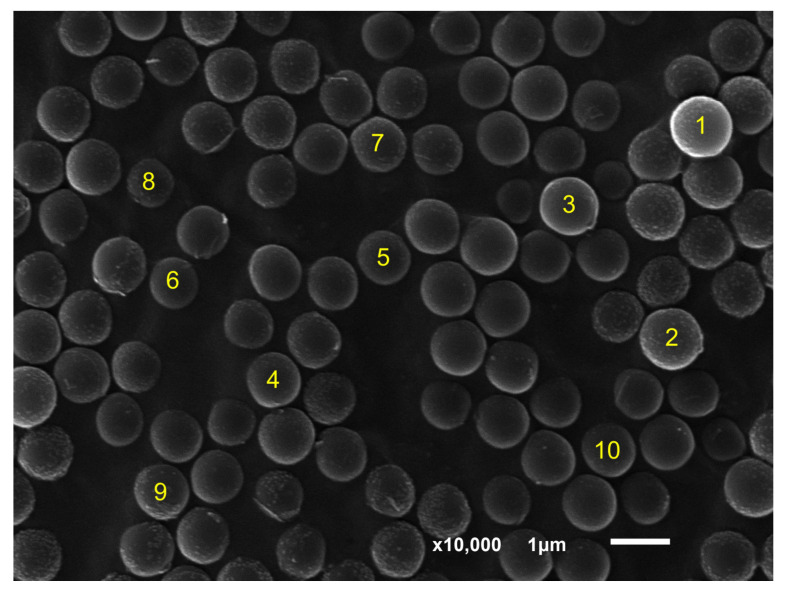
Overview image from scanning electron microscopy (SEM) showing 1 µm PS particles incubated in H_2_O at 37 °C for 76 h. Particles were captured at ten-thousandfold magnification.

In addition, PS particles were incubated in 10% KOH, and their alteration was investigated with scanning electron microscopy (SEM). In order to obtain an overview of the appearance and behavior of the particles that adhered to the carbon tape, 1 µm PS plastic particles incubated in H_2_O for 76 h at 37 °C were examined at ten-thousandfold magnification. It seemed that the particles did not all have the same size, due to the fact that they were located in different plains. Figure 5 shows SEM views of the 1 µm PS microplastic particles, in which ten different particles (yellow numbers 1–10) were selected and measured. The particle size range was 0.94 ± 0.07 µm and the particles were spherical with smooth surfaces (Figure 5). It was found that both larger particles and smaller particles were captured in the image (Figure 5). The main focus was probably on the particle with localization number 3 (Figure 5).

**Figure 6:**
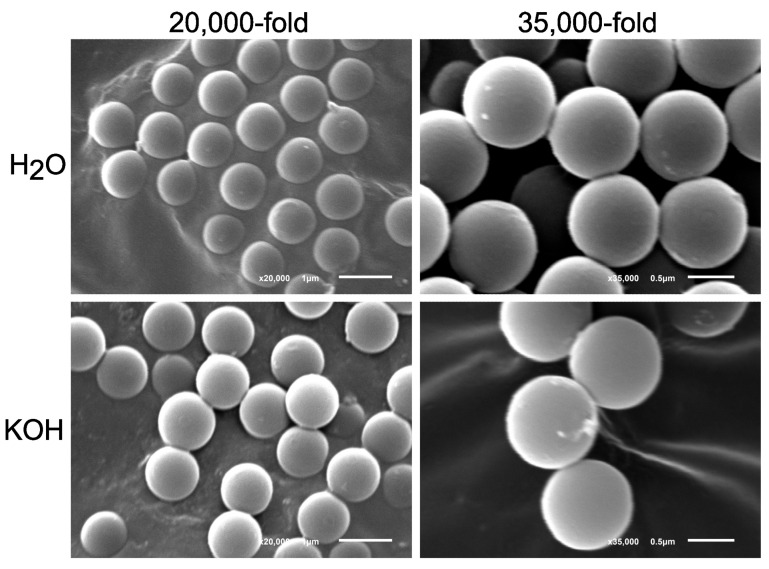
Scanning electron microscopy (SEM) of 1 µm particles after 76 h of incubation at 37 °C in H_2_O (**top**) and in 10% KOH solution (**bottom**) and representative images of 1 µm PS particles with twenty-thousandfold (**left**) and thirty-five-thousandfold (**right**) magnification.

To investigate potential surface or size alterations in 1 µm PS particles after 76 h incubation in 10% KOH at 37 °C, SEM was conducted. The images of the KOH-treated particles were compared with images of those that underwent incubation in H_2_O. Several images of both experimental setups, each with a sample size of 4 (*n* = 4), were captured. Figure 6 displays a representative subset of these images, taken at magnifications of twenty-thousandfold and thirty-five-thousandfold. There were no differences between the H_2_O and the KOH solution (Figure 6). The size distributions of the particles were similar in both solutions, and the surfaces of the particles did not indicate any alterations in either case (Figure 6).

In order to check whether an increase in KOH would yield better digestion progress, the tissue-to-KOH ratio was increased to 1:8 (*w*/*v*) according to a digestion protocol set by Ragusa et al. [18].

**Figure 7:**
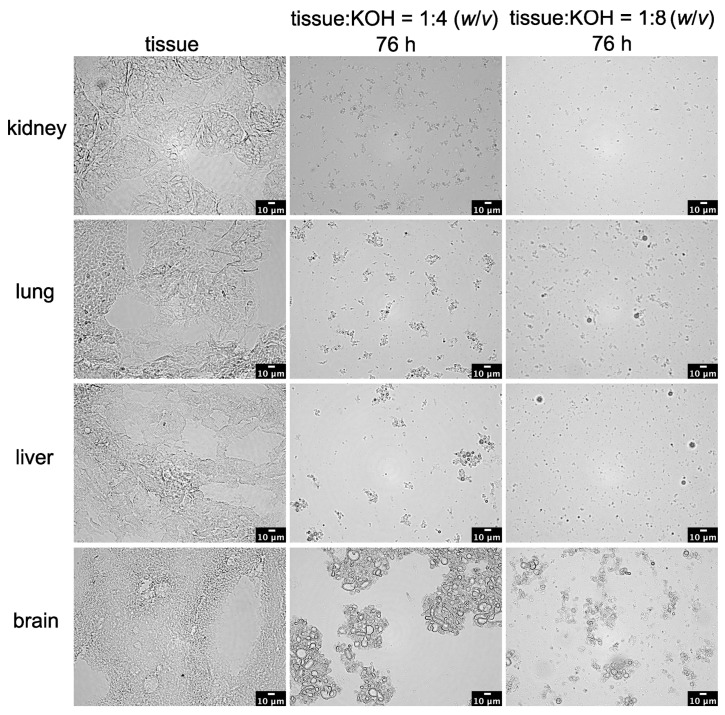
Alkaline digestion of four different tissue samples using 10% KOH solution at an incubation temperature of 37 °C. These representative microscopic images show the comparison between tissue sections (20 µm) of the kidney, lung, liver, and brain and digestion using tissue-to-KOH ratios of 1:4 (*w*/*v*) and 1:8 (*w*/*v*), respectively, after 76 h.

**Figure 8:**
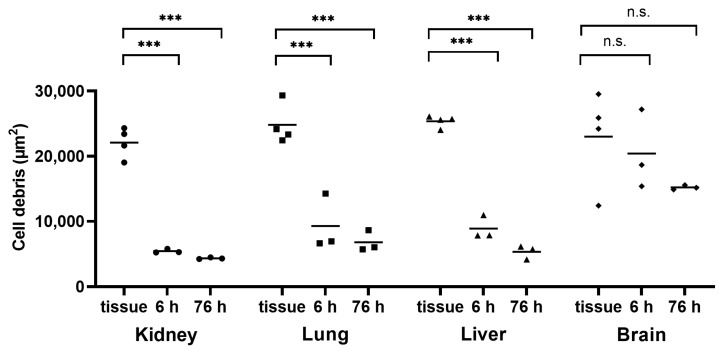
Time-dependent alkaline digestion of tissue samples using 10% KOH solution at a ratio of 1:8 (*w*/*v*) and an incubation temperature of 37 °C. This graphic illustration shows kidney, lung, liver, and brain tissue digestion. The results for the 1:8 dilution ratio were adjusted to the 1:4 dilution ratio used in the previous experiment. Cell debris was detected with light microscopy and subsequent image analysis was performed with a Fiji macro. Data points represent experimental repetition and the crossbar represents the mean. *n* = 4. Statistical significance was determined using a one-way analysis of variance with Dunnett’s multiple comparison test for within-group comparisons (20 µm thickened cryostat versus 6 h and 76 h incubation). n.s.: not significant; ***: *p* < 0.001.

As depicted in Figure 7, the 1:8 (*w*/*v*) tissue-to-KOH ratio appeared to improve the digestive progress in all four tissues when compared with the 1:4 (*w*/*v*) ratio. However, when the dilution factor was included in the calculation, no improvement in the progress of digestion could be seen in any of the tissues (Figure 8). Figure 8 further shows that the significances remained the same as in Figure 3 with the tissue-to-KOH ratio of 1:4 (*w*/*v*). Even for the brain tissue, no improvement in digestion could be seen (Figure 3 and Figure 8).

**Figure 9:**
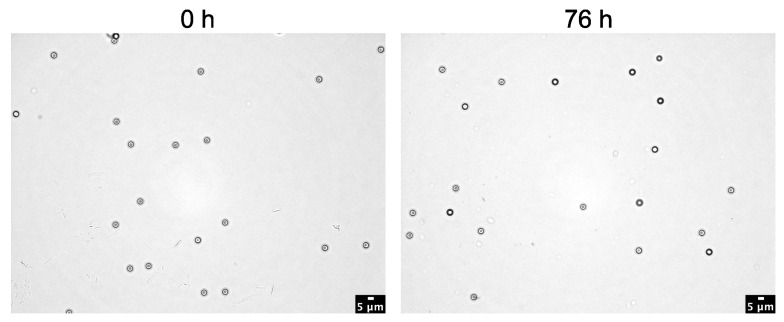
Light microscopic images of the 5 µm particles incubated in water instead of kidney tissue along with 10% KOH at a ratio of 1:8 (*w*/*v*). These representative images were taken after an incubation period of 0 h to 76 h.

To investigate potential alterations in 5 µm PS particles under varying concentrations of KOH, the particles were subjected to incubation in water instead of tissue, along with a 10% KOH solution, at a ratio of 1:8 (*w*/*v*). No changes in the number of particles could be detected between the observation times of 0 h (examination directly after the addition of particles) and 76 h (Figure 9). Furthermore, the figure shows that there were no changes in the surfaces or shapes of the particles.

## 4. Discussion

The aim of this study was to identify a suitable method for effectively digesting various animal tissues to enable subsequent microplastic determination and analysis. Various digestion reagents and protocols were employed to establish a simple and effective method for digesting animal tissue.

The successful application of proteinase K digestion for the isolation and quantification of microplastics in marine organisms has been demonstrated in a marine environmental study [13]. By using this digestion method, Cole et al. were able to overcome the challenge of isolating microplastics from complex, biota-rich seawater samples and marine organisms by achieving a digestion efficiency of >97%. In the course of this study, a digestion protocol that has already been established for human blood samples was used [16]. However, problems were encountered when this protocol was applied to different types of porcine tissue, even though satisfactory digestion results were obtained when the same digestion protocol was applied to human blood samples as a control experiment. This limitation impacted the efficacy of assessing the behavior of plastic particles within the digestive solution. Possible reasons may include that the incubation time and proteinase K concentration used in this experiment were not sufficient for optimal digestion. Furthermore, employing a comprehensive cellular-level homogenization technique or extending the denaturation period before adding proteinase K could potentially yield improved results. However, a publication by Liang et al. offers a potential solution of using proteinase K in a different buffer solution than Tris-HCl, which might give better results [29]. Another study has described optimum digestion for freshwater snails using a mixture of Tris-HCl, proteinase K, and potassium hydroxide (KOH) [30]. The inclusion of KOH can enhance digestion efficiency by facilitating the dissolution of organic matter, leading to improved recovery of microplastics. Additionally, it has been described that these mild digestion conditions do not alter contained microplastics, and therefore, this method is also well-suited for extracting microplastics from tissue [30].

While various digestion protocols have been successfully applied for proteinase K, there is currently no established protocol for organ tissue digestion using the enzymes pepsin or pancreatin based on the current available knowledge and understanding. However, both pepsin and pancreatin have the potential to be utilized for tissue digestion due to their enzymatic properties and functional relevance in the human digestive system. Pepsin, a proteolytic enzyme produced in the stomach, is essential for the breakdown of proteins during the digestive process [31]. Pancreatin, on the other hand, holds promise for tissue digestion by facilitating the breakdown of various components within the tissue, including proteins, fats, and carbohydrates [32]. The digestion protocol with pepsin and pancreatin used in this study resulted in inefficient tissue digestion. Therefore, it was not possible to isolate plastic particles from the organs for quantitative or qualitative analysis. Further research and experimentation are needed to develop effective and reproducible protocols for tissue digestion with pepsin and pancreatin. Thus, increasing the concentrations of the enzymes used or the subsequent addition of enzymes such as trypsin could improve digestion efficiency. In addition, alternative pepsin and pancreatin solutions with higher enzymatic activity, as indicated by the manufacturer, could be investigated in future studies. However, the implementation of these possible optimizations would involve more work and higher costs. Enzymatic digestion is very promising for the isolation of plastic particles from different samples. Nevertheless, given the fascinating physiological processes in the gastrointestinal tract, it is likely that the experimental conditions, such as the concentration of enzymes, will need to be adapted for each sample type.

As a further digestive solution, 10% potassium hydroxide (KOH) was used because it is a commonly used and very simple and effective digestion method for a wide variety of organic materials, like marine samples, animal tissue, and human tissue [18,33,34,35].

After achievement of the successful dissolution of kidney tissue under the selected test conditions, the digestion of other pig organs was examined. The findings of this study revealed that lung and liver tissue were effectively dissolved using a 10% KOH solution after 76 h at 37 °C, while brain tissue was not. The inefficient dissolution of brain tissue is likely attributed to its high lipid content, which may necessitate the use or addition of organic solvents like methanol/chloroform and should be explored in future investigations [36,37].

KOH is frequently employed in the context of microplastic analysis, wherein thorough examination of the effects of this strong alkaline solution on microplastic particles is to ensure that these digestion conditions do not affect the structural and chemical characteristics of the plastic particles.

Considering the extensive use of polystyrene (PS) particles found in various studies on microplastics in animals [30,38,39,40,41] and their easy accessibility and availability on the market at a low cost, the effects of a 10% KOH solution on 1 µm PS particles at 37 °C for 76 h were examined. Moreover, the presence of polystyrene particles has been identified in human blood [16], suggesting that plastic particles of this size may also be relevant in human and animal organs. Light microscopy analysis of the particle solution revealed no significant change in the particle count after KOH incubation, while electron microscopy confirmed no alteration in particle size or surface area. These results align with previous findings by Gulizia et al., who observed no impact of KOH on PS particles even at extreme temperatures [42]. However, other studies have reported potential effects of KOH on PS particles under different experimental conditions, recommending, for instance, incubation at a maximum of 40 °C [43]. Additionally, it is important to consider the possibility of interactions with other plastics when utilizing a 10% KOH solution, as previously described [26].

To assess the impact of 10% KOH solution on digestion efficiency, we increased the tissue:KOH ratio from 1:4 to 1:8, as previously employed for placenta dissolution [18]. However, even after consideration of dilution, no significant enhancement in tissue digestion was observed in this study. Furthermore, the quantity of 5 µm PS particles, easily detectable through light microscopy, remained unaffected by the increased amount of KOH solution.

## 5. Conclusions

The findings of this study indicate the need for further optimization of enzymatic procedures with proteinase K and a pepsin–pancreatin combination for tissue sample digestion, which may vary depending on the tissue type. The goal of optimizing and simplifying enzymatic digestion remains important, as it would enable the gentle isolation of plastic particles with varying sizes and compositions.

Additionally, this study demonstrated that complex organ tissues such as the kidneys, lungs, and liver can be effectively dissolved after incubation in a 10% KOH solution. This opens up possibilities for future investigations of the accumulation of plastic particles in these organs. It is particularly noteworthy that 1 µm polystyrene particles remained unchanged under the selected experimental conditions.

Considering the simplicity and cost-effectiveness of using 10% KOH solution for digestion, as well as its successful resolution of three out of four tested sample types, we recommend its application with the necessary further investigation of KOH’s effects on different plastic particles.

## Data Availability

All data and detailed protocols are freely available upon request to the corresponding author.

## Supplementary Materials

The following supporting information can be downloaded at: https://www.mdpi.com/article/10.3390/toxics11090790/s1, Figure S1. Enzymatic digestion of porcine lung, liver and brain samples using proteinase K (Prot. K). The images depict homogenized lung, liver and brain tissue in the digestion solution before and after incubation in proteinase K, as microscopic examination was not feasible; Figure S2. Enzymatic digestion of lung, liver and brain tissue using pepsin and pancreatin. The kidney homogenates were incubated for 4 h in a 2 mg/mL pepsin solution, followed by an additional 24.5 h in a 2 mg/mL pancreatin solution at 37 °C. Representative microscopic images show the progress of digestion after incubation in pepsin and after subsequent incubation in pancreatin.
