# Supplementary material for: Testing of Different Digestion Solutions on Tissue Samples and the Effects of Used Potassium Hydroxide Solution on Polystyrene Microspheres
Source: Toxics. 2023 Sep 19;11(9):790. doi: 10.3390/toxics11090790 (PMC10536618; doi:10.3390/toxics11090790)
Supplement: Supplementary file 1 [file toxics-11-00790-s001.zip › toxics-2549817-supplementary.pdf]

Supplementary materials

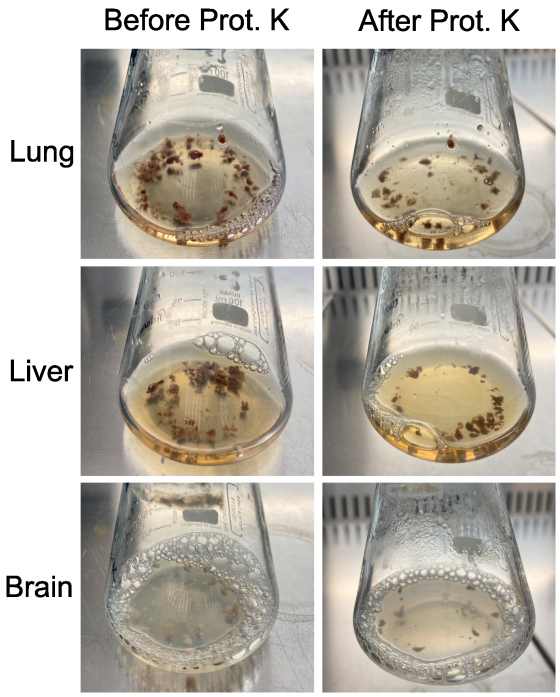

Figure S1. Enzymatic digestion of porcine lung, liver and brain samples using proteinase K (Prot. K). The images depict homogenized lung, liver and brain tissue in the digestion solution before and after incubation in proteinase K, as microscopic examination was not feasible.

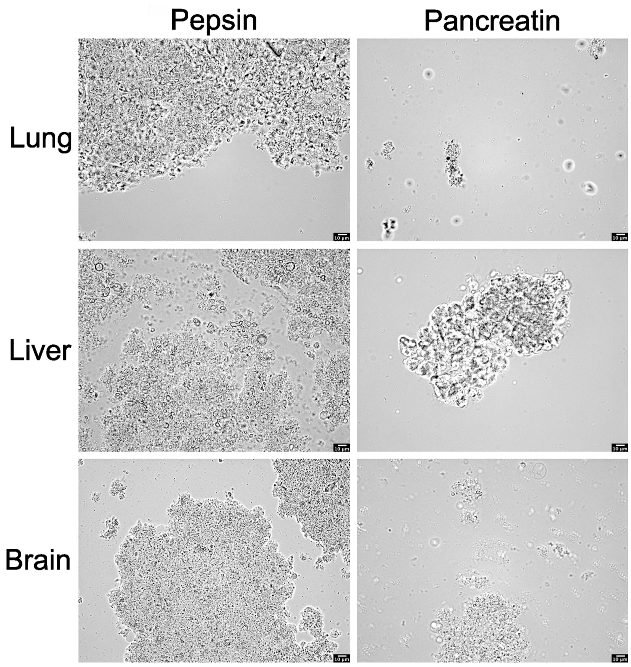

Figure S2. Enzymatic digestion of lung, liver and brain tissue using pepsin and pancreatin. The kidney homogenates were incubated for 4 h in a 2 mg/mL pepsin solution, followed by an additional 24.5 h in a 2 mg/mL pancreatin solution at 37 °C. Representative microscopic images show the progress of digestion after incubation in pepsin and after subsequent incubation in pancreatin.
